# MiR-940 promotes malignant progression of breast cancer by regulating FOXO3

**DOI:** 10.1042/BSR20201337

**Published:** 2020-09-10

**Authors:** Huayao Zhang, Jingwen Peng, Jianguo Lai, Haiping Liu, Zhiyuan Zhang, Xiangdi Li, Baozhen Liang, Xuejun Chen, Baojia Zou, Siyuan Lin, Lihua Zhang

**Affiliations:** 1 Breast thyroid surgery department, SSL Central Hospital of Dongguan City, Dongguan 523326, Guangdong Province, China; 2 Department of Rehabilitation Medicine, Sun Yat-sen Memorial Hospital, Sun Yat-sen University, Guangzhou 510120, Guangdong Province, China; 3 Guangdong Provincial Key Laboratory of Malignant Tumor Epigenetics and Gene Regulation, Sun Yat-sen Memorial Hospital, Sun Yat-sen University, Guangzhou 510120, Guangdong Province, China; 4 Department of Breast Cancer, Cancer Center, Guangdong Provincial People’s Hospital, Guangdong Academy of Medical Sciences, Guangzhou 510000, Guangdong Province, China; 5 Department of Hepatobiliary Surgery, The Fifth Affiliated Hospital, Sun Yat-sen University, Zhuhai 519000, Guangdong Province, China

**Keywords:** Breast cancer, FOXO3, miR-940

## Abstract

Breast cancer (BC) is a common cancer with poor survival. The present study aimed to explore the effect of miR-940 on the process of BC cells and its target gene FOXO3. The expression of miR-940 was assessed in BC tissues and cells using qRT-PCR. Furthermore, the correlation between miR-940 and prognosis of BC patients from the TCGA database was analyzed. CCK8 assays and colony formation assays were used to explore the effect of miR-940 on BC cell proliferation. The invasion abilities were detected by transwell assays. Luciferase reporter assay was performed to scrutinize the relationship between miR-940 and FOXO3. Finally, rescue experiments were performed through FOXO3 down-regulation and miR-940 inhibitors by using CCK8 assays, colony formation assays and transwell assays. miR-940 was significantly up-regulated in BC cells and tissues. In addition, the high level of miR-940 correlated with poor survival of BC patients (*P*=0.023). CCK8 assays, colony formation assays and transwell assays indicated that miR-940 promoted the proliferation and invasion abilities of BC cells. The luciferase reporter assay suggested that miR-940 directly targeted FOXO3. Moreover, we found that the effect of si-FOXO3 was rescued by miR-940 inhibitors in BC cells. miR-940 may promote the proliferation and invasion abilities of BC cells by targeting FOXO3. Our study suggested that miR-940 could be a novel molecular target for therapies against BC.

## Background

Breast cancer incidence ranks the first among women malignancies all over the world [1]. Although the treatment for BC, including surgery, radiation therapy, chemotherapy and endocrine therapy have been improved, BC is still the first killer threatening the health of women [2]. The molecular mechanism involved in the progression of BC is still unclear, and there are challenges in targeting effective biomarkers for therapies against BC. Therefore, it is necessary and important to further investigate the molecular mechanism of BC progression and to identify the desired biomarkers of BC.

MicroRNA (miRNA), a small noncoding RNAs with approximately 22 nucleotides, has been reported to directly bind to the 3′-untranslated regions (3′UTR) of target mRNAs, leading to the repression of translation or induction of degradation of downstream mRNAs [3,4]. Increasing evidences have reported that miRNAs were related to the prognosis of various cancers, and they might play important roles in cell biological behaviors of BC. For example, miR-106b-5p contributes to the lung metastasis of breast cancer via targeting CNN1 and regulating Rho/ROCK1 pathway [5]. miR-137 alleviates doxorubicin resistance in breast cancer through inhibition of epithelial–mesenchymal transition by targeting DUSP4 [6]. Therefore, miRNA might be a promising biomarker for BC.

MiR-940 is an important miRNA that involved in various cancers. In previous studies, the tumor-suppressing role of miR-940 has been reported [7]. However, several studies also demonstrated its oncogenic role [8,9], and the relationship between miR-490 and BC is still unclear. In the present study, we found that miR-940 was significantly up-regulated in BC cells and tissues, and the high level of miR-940 correlated with poor survival of BC patients. Moreover, miR-940 promoted the proliferation and invasion abilities of BC cells by targeting FOXO3. Our study suggested that miR-940 could be a novel molecular target for therapies against BC.

## Methods

### Ethical statement and tissue collection

The fresh BC tissues and normal tissues were obtained from patients who had undergone surgery at the SSL Central Hospital of Dongguan City. Thirty-five pairs of tumor and adjacent tissue specimens were frozen immediately and stored at −80°C. The Ethics Committee approved this study of the SSL Central Hospital of Dongguan City. Informed consent from all patients was obtained in this research.

### Total RNA extraction and qRT-PCR

Total RNA from tissues and cells was extracted using RNAiso Plus (TaKaRa Japan). We measured the concentration of the RNA samples using a NanoDrop 2000 (Thermo Scientific, Wilmington, DE, U.S.A.). A miRNA First Strand cDNA Synthesis Kit (SangonBiotech, China) was used for reverse transcription of miRNA, and the reverse transcription of mRNA was performed with PrimeScript RT Master Mix (TaKaRa, Japan). The cDNA was subjected to real-time PCR on a LightCycler® 96 System (Roche, Switzerland). GAPDH gene was used as an internal control for mRNA, while the expression of miRNA was normalized to small nuclear U6. We analyzed the sample in 10 μl reaction volume in triplicate. PCR amplification consisted of an initial denaturation at 95 °C for 30 s, followed by 42 cycles of amplification at 95 °C for 5 s and 60 °C for 20 s. miR-940 primers used in the present study were as follows: F: 5′-CCTGTCTTACTTTTCCG AAGGAC-3′, R 5′-TTGCTGTATTGTTGCCCATGT-3′; U6 F: 5′-CTCGCTTCGGCAGCACA-3′, R: 5′-AACGCTTCACGAATTTG CGT-3′ .The relative expressions were calculated with the 2^−∆∆CT^ method.

### Cell culture

BC cells (T47D and MCF-7 cell lines) were purchased from ATCC (Manassas, VA, U.S.A.). The BC cells were cultured in DMEM (Gibco; Thermo Fishier Scientific, Suzhou, China) with 10% fetal bovine serum at 37°C in a humidified atmosphere with 5% CO_2_.

### Transfection

T47D and MCF-7 cells were seeded in six-well plates to 60% confluence before transfection. miRNA mimics, inhibitors, siRNAs or overexpressing vectors were transfected using a Lipofectamine 2000 transfection kit (Invitrogen, U.S.A.) according to the manufacturer’s instructions.

### Cell proliferation assay

The proliferation of miR-940 of BC cells was investigated by CCK8 assays. After transfection, BC cells were reseeded into 96-well plates at a density of 4 × 10^3^/well and cultured for 24, 48, 72 and 96 h. The cell viability was determined using a Cell Counting Kit-8 (CCK-8; ImmunoWay Biotechnology Company Plano, TX, U.S.A.), and OD450 values were monitored. We used colony-forming assays to assess the clonogenic ability of BC cells. The treated T47D and MCF-7 cells were seeded into six-well plates at a density of 1000 per well, and incubated at 37°C in a 5% humidified atmosphere for about 2 weeks. When the colonies were visible, we counted the colonies which were fixed in paraformaldehyde after staining with Crystal Violet.

### Transwell invasion assay

To investigate invasion abilities of BC cells, transwell assays were performed using Transwell chambers (Costar, U.S.A.) precoated with Matrigel according to the manufacturer’s protocol. After transfection, 2 × 10^5^ BC cells suspended in serum-free medium were added to the upper chambers (pore size, 8 μm; Corning Inc., Tewksbury, MA, U.S.A.). DMEM with 10% fetal bovine serum was applied to the bottom chambers. After incubating the BC cells for 24 h at 37°C in a 5% humidified atmosphere, the BC cells invaded into the lower membrane surface were fixed in 4% paraformaldehyde. We counted the invaded cells stained with a Crystal Violet staining solution in three randomly selected fields.

### Luciferase reporter assay

FOXO3 sequences containing wild-type (WT-Type) or mutated (Mut-Type) miR-940 binding sites were synthesized and inserted into luciferase vectors, respectively. Then, 293T cells were seeded into 24-well plates at the density of 3 × 10^4^ cells/well. After co-transfection with miR-940 mimics and luciferase vectors for 48 h, we evaluated the Rluc activity with a dual-luciferase reporter assay system (Promega, U.S.A.). With the normalization of luminescence of firefly luciferase, the Renilla luciferase activities were evaluated in triplicate.

### Western blot analysis

RIPA buffer with protease inhibitors (CWBIO, China) was used to lyse the BC cells and extracted the total protein. After separation by SDS-PAGE gels, the proteins were transferred to PVDF membranes. Next, we incubated the protein with primary anti-FOXO3 antibodies (diluted 1:1000, Abcam, China), as well as anti-GAPDH antibodies (diluted 1:1000, ABclonal, China) at 4°C overnight. After the incubation with the secondary antibodies with optimized concentrations, signals with images acquisition were visualized using the Immobilon ECL substrate (Millipore, Germany) and Optimax X-ray Film Processor (Protec, Germany).

### Statistical analysis

In the present study, we acquired the counts of BC miR-940 expression profiles from the Cancer Genome Atlas (TCGA) database in March 2020. BC patients with certain T and N stage were included. Others clinical characteristics including age, T stage, N stage, molecular subtypes, ER status, HER2 status were analyzed in the eligible patients. The BC patients were classified into a low-risk group and a high-risk-group with the median of miR-940 expression.

The relationship between miR-940 expression and clinical characteristics was analyzed by the Chi-square test. Overall survival (OS) was assessed by a Kaplan–Meier analysis and compared by a log-rank test. We used univariate and multivariate Cox proportional hazards regression model to analyze the independent prognosis factors. *P*<0.05 was considered statistically significant. Data analyses were performed using PRISM (GraphPad Software Inc., San Diego, CA, U.S.A.) and Stata version 13.1 (StataCorp., College Station, TX).

## Results

### MiR-940 is up-regulated in BC and related to poor survival

**Figure 1:**
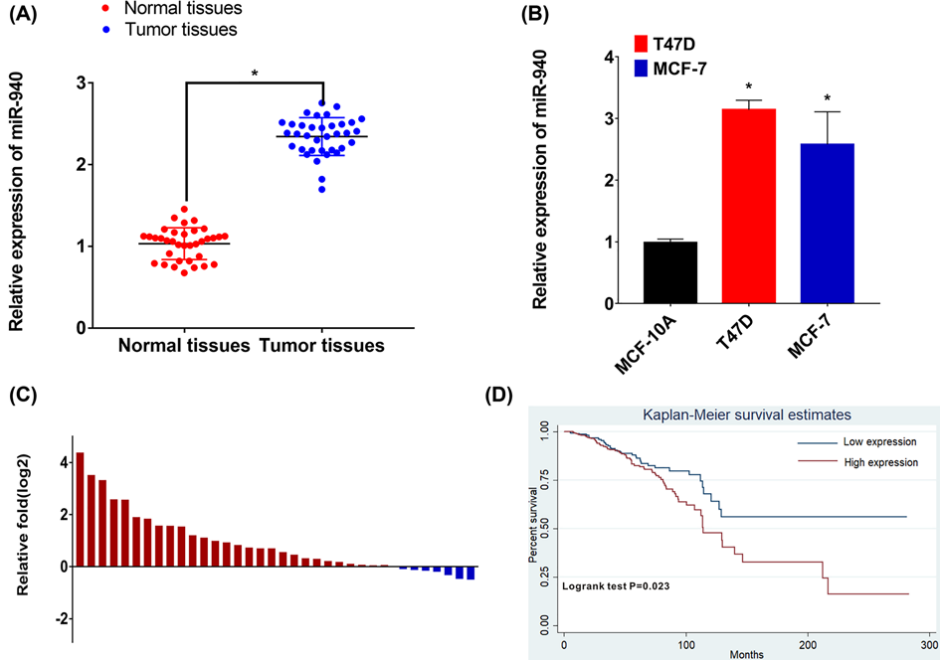
MiR-940 was up-regulated in BC tissues and related to poor patients’ prognosis (**A**) MiR-940 expression was assessed in cancer tissues and normal tissues. (**B**) MiR-940 expression was assessed in BC cells. (**C**) MiR-940 was high in 80% of BC patients. (**D**) Kaplan–Meier’s analyses of correlations between the miR-940 expression and overall survival of the BC patients from TCGA database; BC, breast cancer.

According to the evaluation of miR-940 by qRT-PCR for 35 matched BC tissues and noncancerous tissues, the results indicated that miR-940 was highly expressed in the cancer tissues compared with the noncancerous tissues (Figure 1A). Additionally, the miR-940 levels were high in 80% (28/35) of BC patients (Figure 1C). Moreover, we examined the expression level of miR-940 in BC cells. It was confirmed that miR-940 was increased in T47D and MCF-7 cell lines normalized to MCF-10A cells (Figure 1B).

To further explore the relationship between miR-940 and clinicopathological characteristics, BC patients from TCGA were classified into high and low miR-940 expression groups, and Chi-square test was used to evaluate the clinicopathological characteristics between these two groups. As shown in Table 1, more patients with low miR-940 expression were diagnosed as luminal A subtype (*P*=0.005), and miR-940 seems to have no relationship with clinicopathological characteristics, including age, T stage, N stage, and ER status. Kaplan–Meier survival curve demonstrated that the patient with high miR-940 expression has poor survival compared with the patients with low level (*P*=0.023) (Figure 1D). Next, univariate and multivariate COX regression analyses were performed and showed that the miR-940 expression was an independent prognostic factor for prognosis (HR = 1.51; 95% CI: 1.03–2.21; *P*=0.036) (Table 2). These results indicated that the expression of miR-940 was related to BC progression.

**Table 1:** Baseline characteristics of BC patients from TCGA

Variables	Low expression		High expression		*P*-value
	No.	%	No.	%	
**T stage**					0.065
T1	125	26.48	133	28.12	
T2	274	58.05	291	61.52	
T3	73	15.47	49	10.36	
**N stage**					0.158
N0	221	46.82	228	48.20	
N1	168	35.59	162	34.25	
N2	44	9.32	58	12.26	
N3	39	8.26	25	5.29	
**Subtype**					0.005
Luminal A	292	61.86	234	49.47	
Luminal B	49	10.38	68	14.38	
HER2+	11	2.33	14	2.96	
TN	55	11.65	71	15.01	
Na	65	13.77	86	18.18	
**ER status**					0.096
Negative	87	18.43	111	23.47	
Positive	369	78.18	341	72.09	
Na	16	3.39	21	4.44	
**HER2 status**					0.015
Negative	350	74.15	310	65.54	
Positive	60	12.71	83	17.55	
Na	62	13.14	80	16.91	
**Age (years)**	57.94 ± 12.46		57.27 ± 13.33		0.429

Abbreviation: TN, triple negative

**Table 2:** Univariate and multivariate COX proportional hazards regression analyses

Variables	Univariate analysis			Multivariate analysis		
	HR	95% CI	*P*-value	HR	95% CI	*P*-value
**Age**	1.03	(1.01,1.04)	<0.001	1.03	(1.02,1.05)	<0.001
**T stage**						
T1	1					
T2	1.68	(1.06,2.67)	0.027	1.49	(0.92,2.41)	0.107
T3	1.61	(0.89,2.91)	0.119	1.10	(0.57,2.12)	0.778
**N stage**						
N0	1					
N1	2.10	(1.38,3.20)	0.001	2.27	(1.47, 3.51)	<0.001
N2	2.68	(1.84,7.55)	0.001	3.52	(1.95, 6.33)	<0.001
N3	3.73	(1.84,7.55)	<0.001	4.29	(2.01, 9.13)	<0.001
**Subtype**						
Luminal A	1			1		
Luminal B	1.40	(0.74,2.66)	0.300	1.31	(0.68,2.51)	0.415
HER2+	0.53	(0.07,3.83)	0.527	0.36	(0.05,2.65)	0.316
TN	1.75	(1.01,3.02)	0.047	1.86	(1.06, 3.25)	0.029
Na	2.13	(1.37,3.32)	0.001	1.93	(1.23, 3.04)	0.004
**Expression of miR-940**						
Low expression	1					
High expression	1.54	(1.06,2.23)	0.024	1.51	(1.03,2.21)	0.036

Abbreviations: CI, confidence interval; HR, hazard ratio; TN, triple negative.

### miR-940 promotes the proliferation and invasion of BC cells *in vitro*

**Figure 2:**
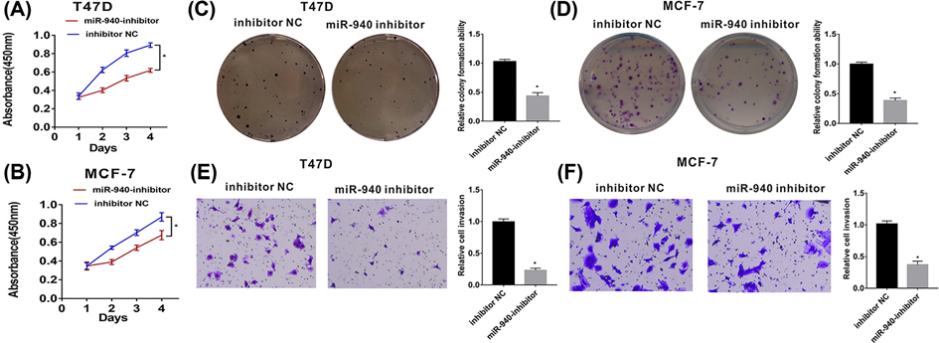
The function of miR-940 in BC cells was assessed by CCK8, colony formation assays and transwell assays (**A** and **B**) Down-regulated miR-940 inhibited the proliferation of BC cells. (**C** and **D**) Down-regulated miR-940 inhibited the colony formation of BC cells. (**E** and **F**) Down-regulated miR-940 inhibited the invasion ability of BC cells; *, *P*<0.05

miR-940 expression was knocked down in T47D and MCF-7 cells treated with miR-940 inhibitors. To explore the functions of miR-940 in cellular proliferation, CCK8 assays were performed. Compared with the control group, miR-940 down-regulation can significantly inhibit BC cells growth (Figure 2A,B). The clonogenic ability of two BC cell lines was determined using colony formation assays, and the results showed that BC cells with down-regulated miR-940 formed a smaller number of colonies compare with control cells (Figure 2C,D). In transwell assays, inhibition of BC cell invasion was showed after knocking down miR-940 in T47D and MCF-7 cells (Figure 2E,F). Taken together, the above results suggested that miR-940 had abilities to promote the proliferation and invasion of BC cells.

### FOXO3 is a direct target of miR-940

**Figure 3:**
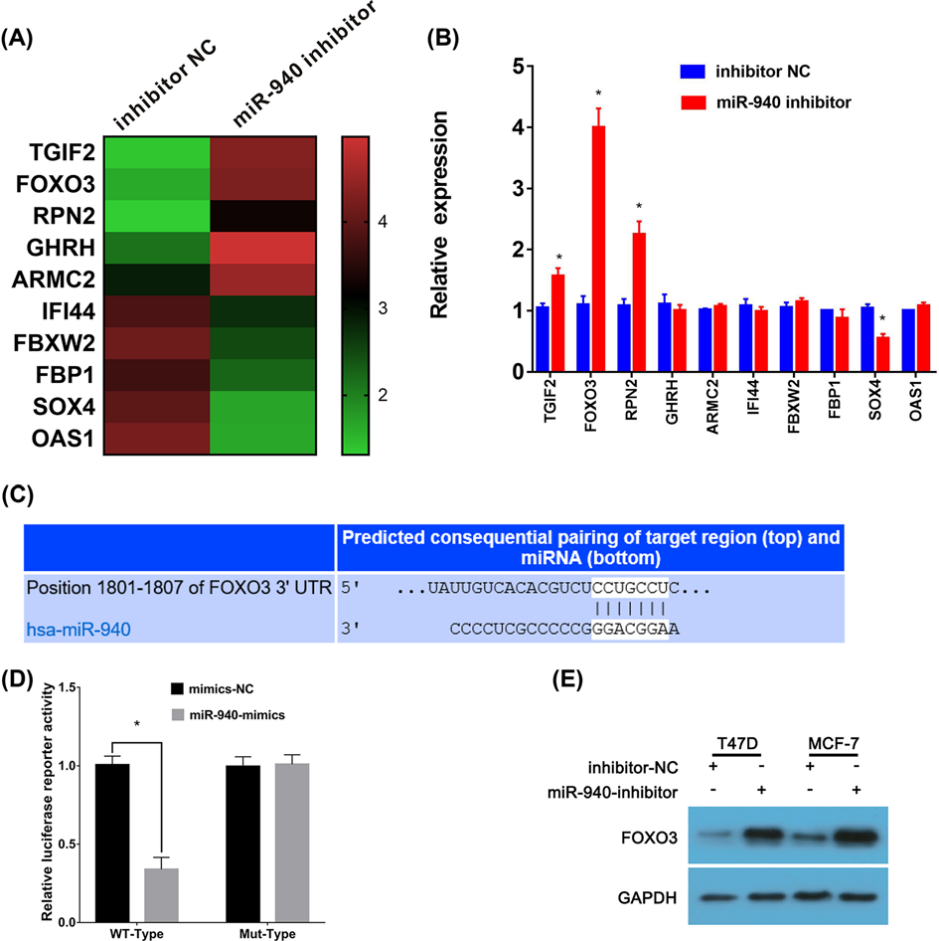
FOXO3 is a direct target of miR-940 (**A**) Top 10 DEGs from RNA-seq were showed. (**B**) The expression of the top 10 DEGs were assessed by qRT-PCR. (**C**) The binding site for miR-940 in the 3-′UTR of FOXO3 predicted by bioinformatics was presented. (**D**) Luciferase reporter assays indicated that the luciferase reporter activity in miR-940 mimics and FOXO3 3′UTR-WT co-transfection group was lower than that in NC-mimics and FOXO3 3′UTR-WT con-transfection group. (**E**) Western blot analyses comparing downregulated-miR-940 BC cells with control cells were shown for FOXO3; BC, breast cancer; *P<0.05.

To investigate the potential molecular mechanism regulated by miR-940 in BC, RNA-seq was performed. We analyzed the differently expressed genes (DEGs) in miR-940 inhibitor-NC group and miR-940-inhibitor group, and listed the top 10 significant DEGs (Figure 3A). Furthermore, these 10 DEGs were evaluated by qRT-PCR, and we found that three DEGs was up-regulated and one was down-regulated by miR-940 inhibitor (Figure 3B). Additionally, the target genes of miR-940 were predicted by TargetScan and miRBase. The results showed that there was a binding site of miR-940 existed in the 3-′UTR region of FOXO3 that was a significant DEGs regulated by miR-940 (Figure 3C). FOXO3, a member of the forkhead type transcription factor family, has been reported to be an important tumor suppressor gene in various human cancers. Therefore, we speculated that miR-940 might regulate biological function via binding FOXO3. To determine whether FOXO3 mRNA is a functional target gene of miR-940, the dual luciferase reporter gene assays was performed. The luciferase activity was decreased in only cells co-transfected with miR-940 mimics and FOXO3-wild type containing the miR-940-binding sequence, while the luciferase activity of the vector with the mutant miR-940-binding site in cells was not affected by miR-940 mimics (Figure 3D). Moreover, the FOXO3 protein level was decreased when the BC cells were transfected with miR-940 inhibitor (Figure 3E). Hence, FOXO2 is one of direct targets of miR-940, and miR-940 may inhibit FOXO3 expression by binding the 3′UTR of FOXO3 in BC cells.

### miR-940 promotes progression of BC by modulating FOXO3

**Figure 4:**
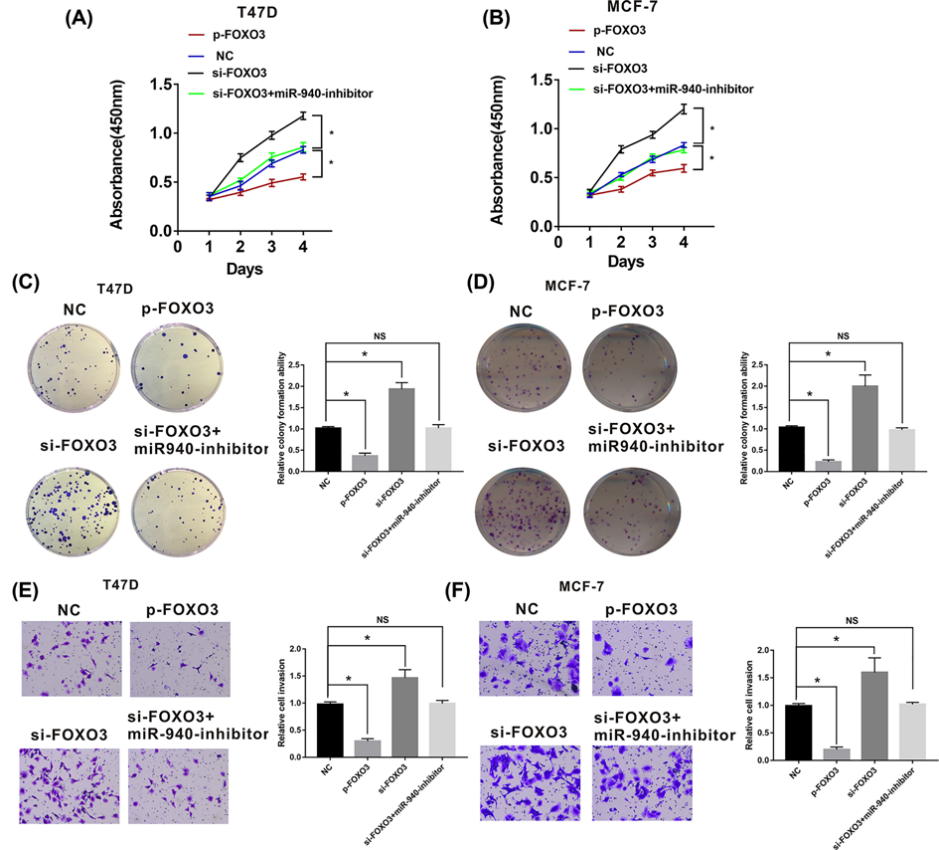
FOXO3 inhibited BC cells development (**A**) and (**B**) CCK8 assays demonstrated that up-regulated FOXO3 inhibited proliferation of BC cells, while FOXO3 down-regulation promoted the proliferation of BC cells. (**C**) and (**D**) Colony formation assays indicated that up-regulated FOXO3 suppressed BC cells growth, while knockdown of FOXO3 enhanced the growth ability of BC cells. (**E**) and (**F**) Transwell assays indicated that overexpression of FOXO3 suppressed BC cells invasion ability, while knockdown of FOXO3 enhanced the invasion ability of BC cells; BC, breast cancer *P<0.05.

Given that miR-940 could directly bind to FOXO3, we speculated that miR-940 promotes progression of BC by modulating FOXO3**.** To test the hypothesis, CCK8 assays, colony-formation assays and transwell assays were performed. The results suggested that the BC cell proliferation was suppressed by the FOXO3 up-regulation and it was promoted by the FOXO3 down-regulation. The rescue assays indicated that the tumor-promoting effect of si-FOXO3 was alleviated by miR-940 inhibitor (Figure 4A–D). The results of transwell assays revealed that the invasion abilities of BC cells were suppressed by up-regulated FOXO3 and were promoted by si-FOXO3. Furthermore, the invasion abilities promoted by down-regulated FOXO3 were restored by miR-940 inhibitor (Figure 4E,F). Taken together, miR-940 promotes proliferation and invasion abilities of BC cells by regulating FOXO3.

## Discussion

In the present study, we identified miR-940 as a highly expressed miRNA in BC tissues and cells. The BC patients with high miR-940 expression from TCGA data have more poor survival than their counterparts. The function approaches *in vitro* revealed that the proliferation and invasion abilities of BC cells were promoted by miR-940. Furthermore, we found that miR-940 could directly bind to the 3-′UTR of FOXO3 and subsequently inhibit the FOXO3 expression. Consistently, these findings revealed a novel molecular mechanism for BC proliferation and invasion.

Currently, although the screen way and development of therapy regimens have significantly improved, BC still has increased rates of morbidity and mortality in female [10]. Numerous studies have indicated that the expression of miRNA is frequently abnormal in BC and related to the cancer proliferation, invasion, apoptosis and chemo-resistance [11–13], suggesting that miRNAs might be promising molecular targets for BC therapies. However, the complex miRNA biological function progression has not been clearly elucidated. As previously reported, miR-940 is thought to related to progression and development in BC. For example, miR-940 induces an osteoblastic phenotype in the bone metastatic microenvironment of BC patients via targeting ARHGAP1 and FAM134A [14]. On the other hand, miR-940 also showed its tumor-suppressing effect. A previous study reported that miR-940 could inhibit cells growth and migration in triple-negative BC [15], which was inconsistent with our results. This discrepancy may be explained by several reasons. First, the BC cells used in present study was not specific triple-negative BC cells, and different cells have different biological characteristics. Second, the data analyzed in the present study was originated from BC patients including four kinds of molecular subtypes. We also found that there was no relationship between miR-940 and the prognosis of patients with triple-negative BC from TCGA in the data that were not shown. Third, this previous study indicated that miR-490 could inhibit BC progression via regulating its target gene ZNF24, while our further experiments showed that miR-940 could promote cancer progression through regulating FOXO3. This discrepancy suggested that miR-940 might play different roles in various subtypes of BC, and we will explore this in future studies.

FOXO3, as a member of the forkhead type transcription factor family [16], is a tumor suppressor often deregulated in different types of human cancers, including prostate cancer, pancreatic cancer and breast cancer [17–19]. It has been reported that FOXOS exerts its function in inhibiting proliferation and promoting apoptosis of cancer cells [20]. When the FOXO3 is activated, it could induce transcription of target genes through binding to the conversed sequence motif GTAAA(C/T)A [21]. In breast cancer, Mahmud et al. demonstrated that EP300 and SIRT1/6 co-regulates Lapatinib sensitivity through modulating FOXO3-acetylation and activity [22]. Another study found that lnRNA LINC01355 could stabilize FOXO3 protein and inhibit the progression of BC [17]. In the present study, using RNA-seq and associated experiments, we found that FOXO3 was regulated by miR-940. Furthermore, luciferase reporter assay suggested that miR-940 could bind directly to the 3′UTR of FOXO3. In rescue experiments, the proliferation and invasion induced by down-regulated FOXO3 could be restored by miR-940 inhibitor, suggesting that miR-940 could promote BC progression by regulating the expression of FOXO3. These results indicated a novel molecular mechanism for BC. However, the precise role of miR-940 and FOXO3 is needed further exploration.

Although we observed some significant results, there were several limitations exist in the present study. First, we analyzed the correlation between miR-940 expression and BC prognosis using the data from the TCGA database. Different institutions may have different methods for miRNA detection and different cutoff of miR-940 value, which means that they lack the normalized standard of miRNAs. Whether the expression of miR-940 is related to the prognosis of BC needs further real-world studies of multicenter. Second, the role of miR-940 was only investigated *in vitro*. Whether miR-940 could promote proliferation *in vivo* is unknown. Therefore, further animal experiments were needed. Third, the present study only explored the functions of miR-940 in promoting proliferation and invasion of BC cells, and miR-940 possibly regulate progression in multiple ways. Hence, the underlying function of miR-940 in BC cells remains unclear, suggesting that detail mechanism needs to be investigated in the development of BC.

## Conclusions

In summary, miR-940 was highly expressed in BC tissues and cells. Additionally, up-regulated miR-940 was related to poor survival in BC patients from the TCGA database. Our results also revealed that miR-940 could promote the proliferation and invasion abilities of BC cells. Furthermore, we verified that miR-940 promotes malignant progression of BC by modulating FOXO3, suggested that miR-940 could be a novel molecular target for therapies against BC.

## Data Availability

The datasets used and/or analyzed during the current study are available from the corresponding author on reasonable request.

## Abbreviations

BC: breast cancer
CI: confidence interval
HR: hazard ratio
OS: overall survival
TN: triple negative

## References

[1] Ferlay J., Soerjomataram I., Dikshit R., Eser S., Mathers C., Rebelo M. et al. (2015) Cancer incidence and mortality worldwide: sources, methods and major patterns in GLOBOCAN 2012. Int. J. Cancer 136, E359–E386 10.1002/ijc.29210 25220842

[2] Wu Q., Jin H., Yang Z., Luo G., Lu Y., Li K. et al. (2010) MiR-150 promotes gastric cancer proliferation by negatively regulating the pro-apoptotic gene EGR2. Biochem. Biophys. Res. Commun. 392, 340–345 10.1016/j.bbrc.2009.12.182 20067763

[3] Lai E.C. (2002) Micro RNAs are complementary to 3′ UTR sequence motifs that mediate negative post-transcriptional regulation. Nat. Genet. 30, 363–364 10.1038/ng865 11896390

[4] Bartel D.P. (2004) MicroRNAs: genomics, biogenesis, mechanism, and function. Cell 116, 281–297 10.1016/S0092-8674(04)00045-5 14744438

[5] Wang Z., Li T.E., Chen M., Pan J.J. and Shen K.W. (2020) miR-106b-5p contributes to the lung metastasis of breast cancer via targeting CNN1 and regulating Rho/ROCK1 pathway. Aging (Albany N.Y.) 12, 1867–1887 10.18632/aging.102719 PMC7053600 31986487

[6] Du F., Yu L., Wu Y., Wang S., Yao J., Zheng X. et al. (2019) miR-137 alleviates doxorubicin resistance in breast cancer through inhibition of epithelial-mesenchymal transition by targeting DUSP4. Cell Death Dis. 10, 922 10.1038/s41419-019-2164-2 31801953 PMC6892819

[7] Yuan B., Liang Y., Wang D. and Luo F. (2015) MiR-940 inhibits hepatocellular carcinoma growth and correlates with prognosis of hepatocellular carcinoma patients. Cancer Sci. 106, 819–824 10.1111/cas.12688 25940592 PMC4520632

[8] Zhou Z., Xu Y.P., Wang L.J. and Kong Y. (2019) miR-940 potentially promotes proliferation and metastasis of endometrial carcinoma through regulation of MRVI1. Biosci. Rep. 39, 10.1042/BSR20190077 PMC6559375 31085718

[9] Fan Y., Che X., Hou K., Zhang M., Wen T., Qu X. et al. (2018) MiR-940 promotes the proliferation and migration of gastric cancer cells through up-regulation of programmed death ligand-1 expression. Exp. Cell Res. 373, 180–187 10.1016/j.yexcr.2018.10.011 30367831

[10] Harding C., Pompei F., Burmistrov D., Welch H.G., Abebe R. and Wilson R. (2015) Breast Cancer Screening, Incidence, and Mortality Across US Counties. JAMA Intern. Med. 175, 1483–1489 10.1001/jamainternmed.2015.3043 26147578

[11] Fong M.Y., Zhou W., Liu L., Alontaga A.Y., Chandra M., Ashby J. et al. (2015) Breast-cancer-secreted miR-122 reprograms glucose metabolism in premetastatic niche to promote metastasis. Nat. Cell Biol. 17, 183–194 10.1038/ncb3094 25621950 PMC4380143

[12] Cui S., Liao X., Ye C., Yin X., Liu M., Hong Y. et al. (2017) ING5 suppresses breast cancer progression and is regulated by miR-24. Mol. Cancer 16, 89 10.1186/s12943-017-0658-z 28490335 PMC5424299

[13] Li H., Liu J., Chen J., Wang H., Yang L., Chen F. et al. (2018) A serum microRNA signature predicts trastuzumab benefit in HER2-positive metastatic breast cancer patients. Nat. Commun. 9, 1614 10.1038/s41467-018-03537-w 29691399 PMC5915573

[14] Hashimoto K., Ochi H., Sunamura S., Kosaka N., Mabuchi Y., Fukuda T. et al. (2018) Cancer-secreted hsa-miR-940 induces an osteoblastic phenotype in the bone metastatic microenvironment via targeting ARHGAP1 and FAM134A. Proc. Natl. Acad. Sci. U.S.A. 115, 2204–2209 10.1073/pnas.1717363115 29440427 PMC5834702

[15] Hou L., Chen M., Yang H., Xing T., Li J., Li G. et al. (2016) MiR-940 Inhibited Cell Growth and Migration in Triple-Negative Breast Cancer. Med. Sci. Monit. 22, 3666–3672 10.12659/MSM.897731 27731867 PMC5072378

[16] Morris B.J., Willcox D.C., Donlon T.A. and Willcox B.J. (2015) FOXO3: A Major Gene for Human Longevity–A Mini-Review. Gerontology 61, 515–525 10.1159/000375235 25832544 PMC5403515

[17] Ai B., Kong X., Wang X., Zhang K., Yang X., Zhai J. et al. (2019) LINC01355 suppresses breast cancer growth through FOXO3-mediated transcriptional repression of CCND1. Cell Death Dis. 10, 502 10.1038/s41419-019-1741-8 31243265 PMC6594972

[18] Yan H., Li Q., Wu J., Hu W., Jiang J., Shi L. et al. (2017) MiR-629 promotes human pancreatic cancer progression by targeting FOXO3. Cell Death Dis. 8, e3154 10.1038/cddis.2017.525 29072689 PMC5682687

[19] Feng Q., He P. and Wang Y. (2018) MicroRNA-223-3p regulates cell chemo-sensitivity by targeting FOXO3 in prostatic cancer. Gene 658, 152–158 10.1016/j.gene.2018.03.013 29518547

[20] Fu Z. and Tindall D.J. (2008) FOXOs, cancer and regulation of apoptosis. Oncogene 27, 2312–2319 10.1038/onc.2008.24 18391973 PMC2819403

[21] Karadedou C.T., Gomes A.R., Chen J., Petkovic M., Ho K.K., Zwolinska A.K. et al. (2012) FOXO3a represses VEGF expression through FOXM1-dependent and -independent mechanisms in breast cancer. Oncogene 31, 1845–1858 10.1038/onc.2011.368 21860419 PMC3232453

[22] Mahmud Z., Gomes A.R., Lee H.J., Aimjongjun S., Jiramongkol Y., Yao S. et al. (2019) EP300 and SIRT1/6 Co-Regulate Lapatinib Sensitivity Via Modulating FOXO3-Acetylation and Activity in Breast Cancer. Cancers (Basel) 11, 10.3390/cancers11081067 PMC6721388 31357743

